# Multimodal detection of molecular residual disease in high-risk locally advanced squamous cell carcinoma of the head and neck

**DOI:** 10.1038/s41418-024-01272-y

**Published:** 2024-02-26

**Authors:** Enrique Sanz-Garcia, Jinfeng Zou, Lisa Avery, Anna Spreafico, John Waldron, David Goldstein, Aaron Hansen, B. C. John Cho, John de Almeida, Andrew Hope, Ali Hosni, Ezra Hahn, Bayardo Perez-Ordonez, Zhen Zhao, Christopher Smith, Yangqiao Zheng, Nitthusha Singaravelan, Scott V. Bratman, Lillian L. Siu

**Affiliations:** 1 Division of Medical Oncology and Hematology, Princess Margaret Cancer Centre, University Health Network, University of Toronto, Toronto, ON Canada; 2 Princess Margaret Cancer Research Institute, University Health Network, University of Toronto, Toronto, ON Canada; 3 Department of Biostatistics, Princess Margaret Cancer Centre, University Health Network, University of Toronto, Toronto, ON Canada; 4 Department of Radiation Oncology, Princess Margaret Cancer Centre, University Health Network, University of Toronto, Toronto, ON Canada; 5 Department of Surgical Oncology, Division of ENT, Princess Margaret Cancer Centre, University Health Network, University of Toronto, Toronto, ON Canada; 6 Department of Pathology, Princess Margaret Cancer Centre, University Health Network, University of Toronto, Toronto, ON Canada; 7 Neogenomics, Babraham Research Park, Cambridge, UK; 8 Cancer Genomics Program, Princess Margaret Cancer Center, University Health Network, University of Toronto, Toronto, ON Canada

**Keywords:** Predictive markers, Cancer

## Abstract

Up to 30% of patients with locally advanced head and neck squamous cell carcinoma (LA-HNSCC) relapse. Molecular residual disease (MRD) detection using multiple assays after definitive therapy has not been reported. In this study, we included patients with LA-HNSCC (stage III Human Papilloma virus (HPV)-positive, III-IVB HPV-negative) treated with curative intent. Plasma was collected pre-treatment, at 4–6 weeks (FU1) and 8-12 weeks (FU2) post-treatment. Circulating tumor DNA (ctDNA) was analyzed using a tumor-informed (RaDaR®) and a tumor-naïve (CAPP-seq) assay. HPV DNA was measured using HPV-sequencing (HPV-seq) and digital PCR (dPCR). A total of 86 plasma samples from 32 patients were analyzed; all patients with at least 1 follow-up sample. Most patients were stage III HPV-positive (50%) and received chemoradiation (78%). No patients had radiological residual disease at FU2. With a median follow-up of 25 months, there were 7 clinical relapses. ctDNA at baseline was detected in 15/17 (88%) by RaDaR and was not associated with recurrence free survival (RFS). Two patients relapsed within a year after definitive therapy and showed MRD at FU2 using RaDaR; detection of ctDNA during follow-up was associated with shorter RFS (*p* < 0.001). ctDNA detection by CAPP-seq pre-treatment and during follow-up was not associated with RFS (*p* = 0.09). HPV DNA using HPV-seq or dPCR during follow-up was associated with shorter RFS (*p* < 0.001). Sensitivity and specificity for MRD at FU2 using RaDaR was 40% and 100% versus 20 and 90.5% using CAPP-seq. Sensitivity and specificity for MRD during follow-up using HPV-seq was 100% and 91.7% versus 50% and 100% using dPCR. In conclusion, HPV DNA and ctDNA can be detected in LA-HNSCC before definitive therapy. The RaDaR assay but not CAPP-seq may detect MRD in patients who relapse within 1 year. HPV-seq may be more sensitive than dPCR for MRD detection.

## Background

Locally advanced head and neck squamous cell carcinoma (LA-HNSCC) is treated with multi-modality therapy consisting of surgery followed by adjuvant radiation (RT) or chemoradiation (CRT); or definitive RT or CRT [1–3]. Despite aggressive treatment, outcomes are poor, particularly for stage III human papilloma virus (HPV)-positive and stage III–IV HPV-negative cases [4], which are associated with a substantial risk of distant metastases (25–30% at 3 years) [5]. Predicting early relapse in order to identify high-risk patients is an important clinical consideration.

Molecular residual disease (MRD) detection has emerged as a biomarker preceding relapse in many tumor types [6–11]. MRD is usually defined as the detection of molecular evidence of cancer using cancer-specific biomarkers, such as circulating tumor DNA (ctDNA), after completion of definitive therapy. Different strategies have been examined in this setting [12]. One approach is the use of tumor-informed ctDNA assays that involve tumor whole exome/genome sequencing to identify patient-specific alterations that enable the design of a personalized assay to track plasma ctDNA [13, 14]. However, tumor sample availability is a limitation. A tumor-naïve approach such as CAPP-Seq (CAncer Personalized Profiling by deep Sequencing), which employs a fixed panel enriched for recurrent mutations of a specific tumor type, could be an appealing alternative [15]. Likewise, targeting viral sequences could allow for tumor-naïve detection of HPV DNA in HPV-positive cases.

Mutation-based ctDNA has been analyzed in LA-HNSCC using plasma and saliva, with its impact in predicting clinical relapse still emerging [16, 17]. Detection of MRD in plasma of HNSCC patients treated with surgery using a tumor-informed ctDNA assay (the RaDaR® assay, Neogenomics Laboratories, Inc) was shown to precede relapse [18]. CAPP-Seq could detect ctDNA pre-treatment in surgically treated HNSCC patients [19]. However, the value of these assays in patients treated with definitive CRT or RT, which represent more than 50% of high-risk LA-HNSCC cases, is not yet known.

HPV-based ctDNA has garnered recent interest for MRD detection in HPV-positive oropharynx cancer (OPC) [20]. Detection of HPV DNA in plasma using digital PCR (dPCR) following CRT is associated with clinical relapse [21–26]. However, dPCR has modest analytical sensitivity and needs to assay separately for each HPV genotype [27]. HPV-sequencing (HPV-seq), involving next generation sequencing (NGS) of viral sequences related to HPV, could overcome these limitations [28].

**Fig. 1:**
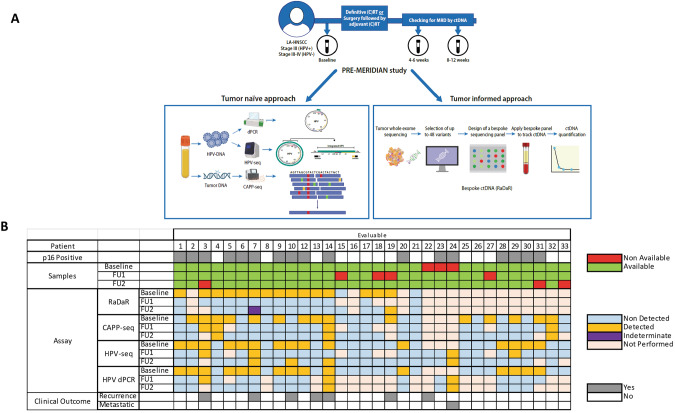
Molecular residual disease detection in locally advanced head and neck cancer (PRE-MERIDIAN) study. **A** Study design and assays included. **B** Samples available and detected by each method and timepoint.

Evaluating these technologies in a single cohort would help to understand their relative merits. We hypothesize that ctDNA using different assays can be detected before definitive therapy and in the immediate follow-up in high risk LA-HNSCC (Fig. 1A). We aim to correlate the detection of ctDNA with clinical relapse at baseline, and at follow-up (4–6 weeks and 8–12 weeks post-definitive therapy). We also aim to explore whether there is a preferred method and optimal timepoint for MRD detection.

## Methods

### Population

From October 2020 to November 2021, we prospectively enrolled patients with high risk LA-HNSCC treated at the Princess Margaret Cancer Centre (PRE-MERIDIAN, NCT04599309). Stage III HPV-positive and stage III, IVA and IVB HPV-negative tumors from oral cavity, oropharynx, hypopharynx and larynx were eligible. Patients were treated with curative intent: surgery followed by adjuvant (C)RT, definitive RT or CRT. Serial blood samples were collected prior to any therapy (baseline), and during follow-up: at 4–6 weeks (FU1), and 8-12 weeks (FU2) following definitive therapy (Fig. 1A). Radiological assessments were performed in all patients at FU2. Patient characteristics were abstracted from the electronic health record. Written informed consent was obtained for all patients. This study was approved by our Institutional Review board. Written informed consent was obtained for all patients. Data cut-off date for the present analysis was June 30, 2023.

### Tumor sequencing

Archival tissue from patients’ diagnostic biopsy was obtained from their primary tumor or neck lymph nodes. In patients who underwent surgery, the surgical specimen was used. DNA was extracted and whole exome sequencing (WES) was performed as previously described [29]. Median depth of read was 250x. Further details are provided in the supplementary methods.

### Plasma analysis

At each time point, 20 mL of peripheral blood was collected in Streck Cell-Free DNA BCT tubes, and plasma and buffy coat were separated by centrifugation and stored at −80 °C. Extraction details are summarized in the supplementary material.

Two different approaches to detect mutation-based ctDNA were employed: RaDaR and CAPP-seq. Briefly, the RaDaR assay is based upon personalized multiplex PCR amplification of cell free DNA (cfDNA). Tumour-specific variants, identified by WES of the primary tumour, were ranked and prioritized for inclusion in patient specific custom panels targeting up to 48 variants and applied to plasma as previously described [9]. Single nucleotide polymorphisms found by panel sequencing of buffy coat DNA were excluded to reduce the potential impact of germline mutations, mosaicism or variants arising from clonal haematopoiesis of indeterminate potential. Median input was 7700 copies (range 3160–18320). A proprietary statistical model was used to determine ctDNA presence (ctDNA positive) or absence (ctDNA negative). The tumor fraction for each sample was reported as estimated variant allele fraction (eVAF). Samples that were considered negative as per the statistical model but were close to the threshold of detection (eVAF >0.001% and/or 2 mutant molecules in a given sample) were noted as indeterminate.

CAPP-seq employed hybrid capture sequencing with a panel optimized for HNSCC, as previously published [19, 30]. Mutect2 was used to identify both somatic SNVs and Indels on a joint calling of multiple cfDNA samples and a matched germline (buffy coat). Technical sequencing errors and putative SNPs were filtered out by iDES “background polishing” with 12 healthy control samples and using gnomAD allele frequency >0.1% (v2.1), low quality mutations were also removed based on the requests of sequencing depth >100, and functional mutations were selected in the sample for each patient [31]. Further details are summarized in the supplementary material. Somatic mutations called on baseline samples were used for the subsequent analyses in the follow-up samples, and the median VAFs of the selected mutations were summarized to represent the ctDNA abundance.

HPV DNA was analyzed using HPV-seq and dPCR. HPV-seq was performed simultaneously with CAPP-seq. HPV genotype was determined from the baseline plasma sample, and HPV genotype-specific DNA levels were quantified as previously described [28] with a normalization factor of 0.6 to account for inflation of HPV-mapped reads relative to human-mapped reads due to incorporation of dual-stranded HPV hybrid capture baits. dPCR was only performed in OPC patients whose tumors were p16 positive by immunohistochemistry, following previous methodology [27]. All ctDNA and HPV DNA measurements were performed by study personnel blinded to clinical outcomes.

### Statistical analysis

Demographics and clinical characteristics were compared by Wilcoxon rank sum test (continuous variables) and Fisher exact tests (categorical variables). Recurrence free survival (RFS) was measured from the start of treatment until recurrence of cancer or death. One patient who had metastatic disease during definitive treatment was removed from the analysis of RFS. Overall survival (OS) was measured from diagnosis to death. Cox proportional hazards models were fit to estimate hazards of recurrences for each assay. Kaplan–Meier (KM) curves were created for dichotomized ctDNA metrics and differences were tested by log-rank test. All statistical analyses were performed in the R programming language version 4.2.2.

## Results

### Patient characteristics, tissue and plasma availability

A total of 32 patients with at least one follow-up sample were included in the analysis (Fig. 1B, Supplementary Fig. 1). The evaluable population characteristics are summarized in Table 1; median age at diagnosis of 63 years (34–71), mostly male (78%), and evenly distributed between HPV-positive and HPV-negative disease. One patient was found to have distant metastases while still on definitive treatment. The most frequent treatment was definitive CRT (78%). No patients had macroscopic residual disease at the time of radiological and clinical assessment performed at FU2. With a median follow-up of 25 months (range: 5.1–32.0), seven (22%) clinical and radiological relapses were observed (Table 2): three during the first year and four during the second year. 2-year RFS was 76% (95% CI 61–95) and 2-year OS was 94% (95% CI 85–100).

**Table 1:** Characteristics of the evaluable patients in the study (*N* = 32).

Characteristics	Patients (%)
Gender	
Male	25 (78)
Female	7 (22)
Stage	
III HPV positive	16 (50)
III HPV negative	7 (22)
IV A-B HPV negative	8 (25)
IV C HPV positive	1 (3)
Primary site	
Oropharynx	23 (72)
Larynx	5 (16)
Oral Cavity	3 (9)
Hypopharynx	1 (3)
Papilloma Virus (p16)	
Positive	17 (53)
Negative/Not done	15 (47)
Smoking	
Never smoker	7 (22)
Former smoker	7 (22)
Smoker	18 (56)
Alcohol intake	
Never	9 (28)
Former	2(6)
Active occasional-moderate	19 (59)
Active heavy	2 (6)
Treatment	
Surgery	1 (3)
Surgery followed by adjuvant radiation	2 (6)
Surgery followed by adjuvant chemoradiation	1 (3)
Definitive radiation	3 (9)
Definitive chemoradiation	25 (78)
Cisplatin	
Weekly dose (40 mg/m^2^) × up to 7 doses	12 (46)
High dose (100 mg/m^2^) × up to 3 doses	14 (54)
Non applicable	6

Only patients with at least a follow-up sample were included.

*HPV* human papilloma virus.

**Table 2:** Patients with clinical or radiological recurrence among the evaluable population.

Patient ID	19	22	7	3	14	13	10
Primary	Larynx	Oral Cavity	OPC	OPC	OPC	Larynx	OPC
Stage	III	III	III	III	III	III	III
p16	Negative	Negative	Positive	Positive	Positive	Negative	Positive
Treatment	RT	Surgery + RT	CRT	CRT	CRT	CRT	CRT
Time to recurrence (days)	203	228	356	605	638	653	731
Site of recurrence	Local	Local	Distant (lung)	Distant (lung)	Local and distant (lung)	Local	Local
Treatment of recurrence	Salvage surgery	Systemic therapy	None	Lung SBRT	Systemic treatment	Salvage surgery	Systemic therapy
Alive	No	No	No	Yes	Yes	Yes	Yes
RaDaR	**FU2:**+	Not performed	FU1:− **FU2: Indet**.	FU1:−	FU1:− FU2:−	FU1:− FU2:−	FU1:− FU2:−
CAPP-seq	FU2:−	Not evaluable	FU1:− FU2:−	**FU1:**+	**FU1:**+ **FU2:**+	FU1:− FU2:−	FU1:− FU2:−
HPV-seq	FU2: NA	FU1/2: NA	**FU1:**+ **FU2:**+	**FU1:**+	FU1:− **FU2:**+	FU1/2: NA	FU1:− **FU2:**+
dPCR (HPV)	FU2: NA	FU1/2: NA	FU1:− FU2:−	**FU1:**+	**FU1:**+ **FU2:**+	FU1/2: NA	FU1:− FU2:−

Main characteristics at baseline, recurrence and assays available for each patient and follow-up timepoints (bold represents detectable).

*CRT* chemoradiation, *dPCR* digital PCR, *FU* follow-up, *Indet* indeterminate, *NA* non-applicable, *OPC* oropharynx, *RT* Radiation.

Eighty-six plasma samples were collected from 32 patients. Three patients did not have baseline plasma collected. Both follow-up plasma samples were collected in 25 patients; the rest had only one follow-up sample (three only had FU1, four had FU2). One sample was excluded from the CAPP-seq and HPV-seq analyses because it failed quality control using NGSCheckMate [32]. WES in matched tumor tissue was attempted in the first 20 patients due to resource limitations, 3 with insufficient viable tissue; 17 patients (85%) were evaluable for RaDaR. CAPP-seq was analyzed in the 29 patients with baseline sample as MRD was only evaluable using this approach if there was baseline ctDNA detection. HPV-seq was analyzed in all 32 evaluable patients regardless of p16 status; HPV dPCR was applied using genotype-matched assays (i.e., targeting the genotypes, HPV 16 and HPV 35, detected by HPV-seq in this cohort) only in those patients with OPC that had p16 expression by immunohistochemistry in the tumor (*N* = 17) (Fig. 1B and Supplementary Fig. 1).

### Circulating tumor DNA prior to definitive therapy

We first evaluated the detection of tumor-informed ctDNA at baseline (Supplementary Table 1). A total of 15/17 (88%) with WES had detected ctDNA using RaDaR; median eVAF was 0.3% (0–11.1%). The two patients with undetected ctDNA had an oral cavity tumor: Patient 15 had T3N0 and patient 21 had a T4N2 spindle cell squamous cell carcinoma. A higher median eVAF was observed in OPC (median 1.9% versus 0.2% in larynx, 0.001% in hypopharynx and undetectable in oral cavity, p = 0.03). Moreover, p16 positive tumors showed higher median eVAF (2.0% vs. 0.1%, *p* < 0.001), regardless of primary tumor location. Baseline eVAF was not associated with RFS (HR: 1.14; 95% CI 0.85–1.54; *p* = 0.37). In contrast, ctDNA was detected in 15/29 patients (51.7%) using CAPP-seq. This frequency of ctDNA detection by CAPP-seq (9/17, 53%) was similar when limited to the subset of 17 patients who also underwent RaDaR analysis at baseline. However, in comparison to RaDaR, which was successful in ctDNA detection in 88% (15/17) of patients, the baseline detection rate by CAPP-seq is significantly lower (*p* = 0.03). Baseline median VAF was not significantly different by primary tumor location or by p16 status and was not associated with RFS (HR: 0.84; 95% CI 0.38–1.83; *p* = 0.66).

### HPV DNA prior to definitive therapy

HPV DNA was evaluated in all 29 baseline plasma samples using HPV-seq (Supplementary Table 2). Compared with p16 immunohistochemistry on OPC tumors, plasma HPV DNA detection by HPV-seq had 100% sensitivity (15/15 detected among p16 positive) and 100% specificity (0/14 detected among p16 negative). The median number of copies of HPV DNA/mL was 2112.7 (14.5–39904.9); levels were not associated with RFS (HR: 1.00; 95% CI 0.99-1.00; *p* = 0.31). Detected HPV genotypes included HPV 16 (14/15) and HPV 35 (1/15). Among baseline samples also evaluated by HPV dPCR, 15/15 had detectable levels with a median number of copies/mL similar to HPV-seq (2084.8; range 16.9–29957.6).

### Circulating tumor DNA to predict recurrence

**Fig. 2:**
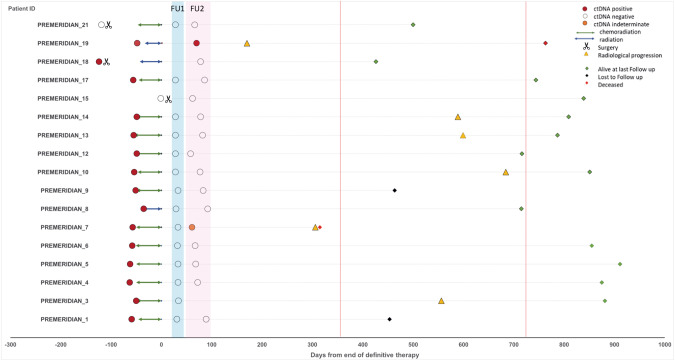
Patients with baseline and follow-up samples using bespoke ctDNA (RaDaR^TM^) assay. ctDNA circulating tumor DNA, FU follow-up.

**Fig. 3:**
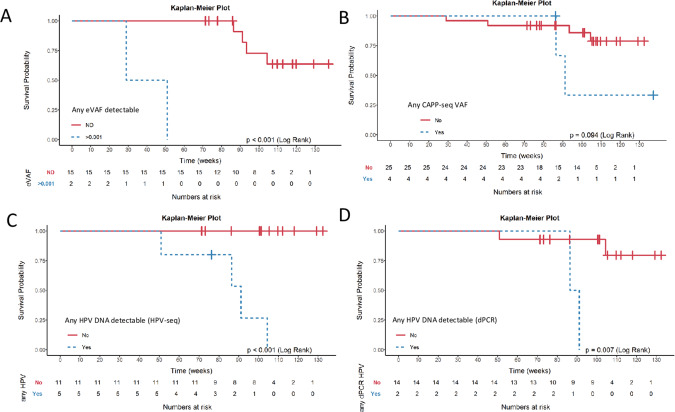
Relapse-free survival according to ctDNA or HPV DNA detection by each method for both follow-up timepoints. **A** RaDaR **B** CAPP-seq **C** HPV-seq **D** digital PCR. Note that for the RaDaR assay the indeterminate result has been considered positive. For CAPP-seq also patients with undetectable plasma at baseline have been included and considered not detected in follow-up.

We then examined the detection of ctDNA during follow-up. Detection of ctDNA during follow-up according to the RaDaR assay was observed in 1/30 samples (3.3%) (Patient 19). That plasma sample was taken at FU2 and had an eVAF of 0.004%. Another patient’s FU2 plasma sample was close to the threshold to be considered positive by RaDaR with an eVAF of 0.001% and was thus called as indeterminate (Patient 7). Both patients subsequently relapsed (one with local recurrence, the other with distant disease) during the first year of follow-up (Table 2, Fig. 2); lead time to clinical/radiological progression was 100 and 245 days, respectively. All other FU1 and/or FU2 samples were negative for ctDNA. Four other patients tested for RaDaR during follow-up relapsed beyond the first year of surveillance (Patients 3, 10, 13 and 14), with a median lead time from last sample to recurrence of 517 days (511–607). An eVAF ≥ 0.001% in plasma samples during the initial 12 weeks of follow-up was associated with inferior RFS (Fig. 3A); 2-year RFS was 0% in eVAF ≥ 0.001% vs. 73% (51–100%) in ctDNA negative patients.

We also analyzed MRD detection during follow-up using CAPP-seq in patients with detectable ctDNA at baseline. At FU1, 4/12 (33%) plasma samples showed ctDNA detection, lower at FU2 (3/13, 23%). These three FU2 samples were from patients whose FU1 sample showed ctDNA detection (Patients 4, 14 and 32). Only Patient 14 had clinical recurrence, with an increase in median VAF from FU1 to FU2. Patient 3 only had FU1 sample available that had detected ctDNA and experienced recurrence. There were two other patients with detection of ctDNA during follow-up who have not recurred (Patient 4 and 32). Detection of ctDNA using CAPP-seq during follow-up was not associated with RFS (Fig. 3B): 2-year RFS was 33% (7–100%) in ctDNA positive vs. 86% (72–100%) in ctDNA negative.

### HPV DNA to predict recurrence

HPV DNA was analyzed during follow-up in all 32 evaluable patients. A total of 8 follow-up plasma samples from 6 patients (18.8%) were positive for HPV DNA by HPV-seq including the two follow-up samples from Patient 24 who had metastatic disease during CRT. The remaining 6 follow-up plasma samples with HPV DNA detection were from 5 patients, four of whom experienced relapsed (Patients 3, 7, 10 and 14). Patient 7 was positive in FU1 and FU2, with increasing number of HPV DNA copies. Patients 10 and 14 were positive for HPV DNA at FU2 but were negative at FU1. Patient 3 was positive for HPV DNA in the only available sample (FU1). In contrast, one patient with HPV DNA detection at follow-up (FU1) did not recur: Patient 29. Detection of HPV DNA during follow-up using HPV-seq was associated with RFS in p16 positive OPC patients (Fig. 3C): RFS-2 years was 27% (5–100%) in HPV DNA positive vs. 100% (100–100%) in HPV DNA negative. Similarly, detection of HPV DNA using dPCR was associated with RFS in p16 positive OPC patient (Fig. 3D). However, only 2/4 patients (50%) with recurrence (Patients 3 and 14) had detectable HPV DNA in follow-up samples using dPCR.

### Correlation between different approaches

There was a strong statistically significant correlation at baseline (*r* = 0.61) between eVAF (RaDaR) and median VAF (CAPP-seq) (Supplementary Fig. 2), but that correlation became weaker and non-significant at FU1 and FU2 (*r* = 0.15 and 0.03, respectively) (Supplementary Table 3). HPV-seq and dPCR had a strong statistically significant correlation at baseline (*r* = 0.98), and this correlation retained statistical significance at FU1 (*r* = 0.60) and FU2 (*r* = 0.70). We also compared the different approaches in terms of detecting MRD at FU1 and FU2 (Table 3). Overall, all the assays detected MRD with higher accuracy at FU2. At that timepoint, RaDaR showed a high specificity (100%) but a low sensitivity (40%), while CAPP-seq showed a relatively lower specificity and sensitivity (90% and 20%, respectively). In contrast, detection of HPV DNA using HPV-seq showed the highest sensitivity and specificity (100%) while sensitivity was lower using dPCR (33%). When limiting the analysis only to p16+ OPC, HPV DNA detection using HPV-seq was the most accurate approach to detect MRD with an accuracy of 87.5% at any timepoint and 100% at FU2 (Supplementary Table 4).

**Table 3:** Assay’s performance in detecting MRD in the evaluable population, excluding the patient with metastatic disease during treatment.

	Assay	N	N Relapse	N Positive	N True positive	NPV (%)	PPV (%)	Sensitivity (%)	Specificity (%)	Accuracy (%)
FU1	RaDaR	14	5	0	0	64.3	–	0	100	64.3
	CAPP-seq	24	5	4	2	85	50	40	89.5	79.2
	HPV-seq	15	4	3	2	83.3	66.7	50	90.9	80
	dPCR	15	4	2	2	84.6	100	50	100	86.7
FU2	RaDaR	16	5	2	2	78.6	100	40	100	81.3
	CAPP-seq	26	5	3	1	82.6	33.3	20	90.5	76.9
	HPV-seq	14	3	3	3	100	100	100	100	100
	dPCR	14	3	1	1	84.6	100	33.3	100	85.7
Any FU	RaDaR	17	6	2	2	73.3	100	33.3	100	76.4
	CAPP-seq	29	6	4	2	84	50	33.3	91.3	79.3
	HPV-seq	16	4	5	4	100	80	100	91.7	93.8
	dPCR	16	4	2	2	85.7	100	50	100	87.5
Combination of different assays										
FU1	All Assays	13	4	5	3	77.8	75	60	87.5	76.9
	RaDaR + HPV-seq	13	5	3	3	80	100	60	100	84.6
	CAPP-seq + HPV-seq	24	5	5	2	84.2	40	40	84.2	75
FU2	All Assays	16	5	5	4	90.9	80	80	91	87.5
	RaDaR + HPV-seq	16	5	4	4	91.7	100	80	100	93.8
	CAPP-seq + HPV-seq	25	5	5	3	90	60	60	90	84
Any FU	All Assays	14	6	5	5	87.5	83.3	83.3	87.5	85.7
	RaDaR + HPV-seq	14	6	5	5	88.9	100	83.3	100	92.3
	CAPP-seq + HPV-seq	21	5	7	4	92.3	57.1	80	81.3	81

Patients with a sample not available for analysis were not considered for analysis at the specific follow-up, while a patient with one follow-up sample negative but the other not performed/available, were removed from the analysis at any follow-up.

*FU* follow up, *NPV* negative predictive value, *PPV* positive predictive value.

We performed an exploratory analysis to evaluate MRD detection with 2 methods (CAPP-seq/RaDaR and HPV-seq) or three methods. The best performance in terms of MRD detection was observed with RaDaR + HPV-seq at FU2 with a sensitivity of 80%, specificity of 100% and accuracy of 93.8% (Table 3). We also analyzed 2 year-RFS combining two or three methods (Supplementary Table 5). RFS is shorter in patients with detected ctDNA by RaDaR and/or HPV DNA detection by HPV-seq at FU2 (2 year-RFS 88% vs. 25%, *p* < 0.001). However, interpretation of the results is limited due to low number of patients for each approach.

## Discussion

In this study, we explored the value of ctDNA and HPV DNA pre- and post-definitive therapy in patients with high-risk LA-HNSCC. To our knowledge, this is the first study to examine different ctDNA analytical approaches in this setting. Even though ctDNA is detected in most patients before definitive therapy using a tumor-informed ctDNA assay, its sensitivity is lower for MRD. A tumor-naïve ctDNA assay performed comparatively worse to detect both pre- and post-treatment ctDNA. In contrast, HPV DNA was the most reliable way to detect MRD in HPV-driven tumors, especially HPV-seq.

We showed that 88% of our patients had ctDNA detected before definitive therapy using a bespoke ctDNA assay. This frequency is similar to one prior study (LIONESS) that also used RaDaR [33] and higher than a tumor agnostic targeted NGS assay incorporating 26 genes and HPV 16, where ctDNA was detected in 77% of cases [34]. It is noteworthy that WES was successfully performed in more than 80% of the cases with available tumor specimen, including patients who only had a core biopsy for diagnosis. These results support the feasibility of bespoke ctDNA evaluations in patients with high risk LA-HNSCC who do not get upfront surgery and typically only have small biopsies.

Bespoke ctDNA analysis has shown significant value in predicting clinical recurrence in patients after surgery for colorectal, breast and lung cancers [9, 13, 14] and post definitive CRT in rectum and lung [35, 36]. In HNSCC, the LIONESS study showed that ctDNA detection using same assay as ours, was associated with recurrence after surgery, with a median lead time of 122 days [33]. We only detected 2/6 patients who had subsequent clinical/radiological relapse, despite the positive predictive value (PPV) and specificity of RaDaR being 100% in our cohort. Of note, both patients recurred within less than a year post-treatment suggesting that this approach may only be able to detect the early recurrences. Some differences are noted between both studies. First, our study consisted of patients treated primarily with a non-surgical approach (>75%), while LIONESS included only surgical patients. Second, we included only patients with stage III-IV tumors while LIONESS included 27% of patients with stage I-II. Third, we analyzed only two timepoints, within twelve weeks of definitive treatment completion, compared to a longitudinal approach in LIONESS, which could explain our lower sensitivity was lower and that we only detected recurrences within a year. When restricting in the LIONESS study to samples within 12 weeks post-definitive treatment, sensitivity was nearly identical to our study (38%).

CAPP-seq, a tumor-naïve approach, has been evaluated in lung cancer showing that ctDNA detection after definitive treatment predicted recurrence [36, 37]. In LA-HNSCC, CAPP-seq can detect ctDNA pre-treatment in two thirds of patients, a slightly higher frequency than in this cohort (53%) [19]. We have also previously reported using CAPP-seq that a decrease in ctDNA within 3-4 weeks after administration of chemotherapy or immunotherapy in recurrent or metastatic HNSCC was associated with prolonged survival [38]. In our current analysis, CAPP-seq showed lower sensitivity and specificity and higher numbers of false positive results. Two patients were found to have detected ctDNA at follow-up using CAPP-seq and recurred. For patient 3, FU2 was not available, so it is unclear if ctDNA had subsequently cleared. For patient 14, baseline VAF by CAPP-seq was the lowest among detectable cases while VAF at FU1/FU2 increased from baseline despite no evidence of disease in the radiological assessments at FU2. These results along with a late recurrence (>500 days) suggest a potential false positive, rather than a true MRD result. The different performances could be attributed to variations in the tumor burden (e.g., locally advanced vs. metastatic disease) and/or technical factors related to sequencing design and mutation detection algorithms. As we only tracked variants that were present at baseline, future studies could evaluate the potential added utility of detecting variants that emerge during surveillance.

In our cohort, the highest accuracy for MRD was HPV DNA in p16 positive OPC. HPV-seq outperformed dPCR at both timepoints, especially at 8–12 weeks after definitive treatment. In cervix cancer we had previously reported 100% sensitivity but a lower specificity (67%) using HPV-seq [26]; this lower specificity may have been explained by the timepoint of blood collection (last day of CRT) as opposed to during follow-up. Another dPCR assay (tumor tissue modified viral) has been validated in p16+ OPC in different cohorts [26, 39] with pre-treatment sensitivity of 92.5% and negative predictive value (NPV) of 99.4% [25], but limited accuracy of MRD detection in the first 6 months post-CRT. A recent meta-analysis from several studies has shown a sensitivity of 91% for HPV DNA detection using different HPV genotypes including only plasma samples at diagnosis [40]. The sensitivity in our study at baseline was 100% for both HPV-seq and dPCR. In contrast, that meta-analysis did not include follow-up samples, thus sensitivity for MRD was not assessed. In our cohort, the sensitivity for MRD was 100% for HPV-seq and 50% for dPCR, with only four recurrences reported in p16 positive OPC. The two cases where MRD was detected by HPV-seq but not by dPCR were from patients whose follow-up samples showed low number of HPV copies just at or below the limit of detection of dPCR (approximately 1 copy/ml): patient 7 (FU1 0.28 and FU 1.12 copies/ml) and patient 10 (FU2 0.18 copies/ml). Further evaluation of HPV-seq is needed in larger cohorts to validate the higher accuracy for MRD detection during follow-up compared with dPCR in this patient population.

Recently, a study has evaluated a combined NGS-based tumor-naïve assay targeting 26 genes and HPV-16 in patients with LA-HNSCC within 12 weeks post-treatment [34]. MRD was identified in 41% of patients with detectable ctDNA at baseline. However, the number of recurrences was higher than in our study (44 vs. 22%). Moreover, the combination of HPV-seq with RaDaR from our study outperformed their assay (NPV 88.9% vs. 83.3%, PPV 100% vs. 82.3%, sensitivity 83.3% vs. 77.8%, specificity 100% vs. 86.9%, accuracy 92.3% vs. 79.1% respectively).

We note several limitations. Despite this being a prospective study, the plasma analysis was performed after all patients were recruited. However, research personnel performing the plasma analysis were blinded to clinical outcome. Second, the number of recruited patients and plasma samples are lower than expected, partially due to limitations to recruitment and plasma biobanking imposed by COVID restrictions. Moreover, all tests could not be performed in all samples, reducing the validity of our conclusions when making comparisons across the assays. The small sample size in our study limits the potential applicability of our findings without further validation in larger studies. Finally, this study was not powered to evaluate the potential value of ctDNA kinetics in the immediate post-treatment setting [41].

In conclusion, a tumor-informed assay and viral DNA (in HPV-driven tumors) can detect ctDNA before definitive therapy in patients with high-risk LA-HNSCC. We proposed that MRD can be better captured at 8–12 weeks post-definite therapy at least in patients treated with definitive RT or CRT. Among all the assays, HPV DNA detection (specially HPV-seq) performs best in terms of detecting MRD at 8–12 weeks post-definitive therapy. However, tumor-informed ctDNA can also detect MRD in patients that relapse within a year of treatment. Further validation of these results is ongoing in a prospective multicentric investigator-initiated study (MERIDIAN, NCT05414032), that includes a cancer interception strategy by randomizing patients who have MRD at FU2 to a novel bispecific checkpoint inhibitor (AZD2936) or observation. This study will aim to complete recruitment end of 2025 with an expected readout in 2026. The PRE-MERIDIAN study has informed the design of MERIDIAN serving as an important feasibility pilot cohort to evaluate MRD in LA-HNSCC.

### Supplementary information

Supplemental Material

## Data Availability

All the clinical and molecular data can be shared de-identified at the publication of the manuscript under request to the corresponding author (Lillian Siu).
