# Supplementary material for: Multimodal detection of molecular residual disease in high-risk locally advanced squamous cell carcinoma of the head and neck
Source: Cell Death Differ. 2024 Feb 26;31(4):460–8. doi: 10.1038/s41418-024-01272-y (PMC11043441; doi:10.1038/s41418-024-01272-y)
Supplement: Supplementary file 1 — Supplemental Material [file 41418_2024_1272_MOESM1_ESM.pdf]

Supplementary Methods

Tumor sequencing details

All samples that were successful for WES met the recommended tumor mean coverage (180x) and uniformity (>70% of target bases >100x deduplicated coverage) metrics. Matched normal WES quality control was also successful in meeting the recommended metrics of mean coverage (50x) and uniformity (>70% of target bases >30x deduplicated coverage)

Plasma analysis

Cell Free DNA was extracted with the QIAamp Circulating Nucleic Acid Kit (QIAGEN). Buffy coat genomic DNA was extracted with the DNeasy Blood & Tissue Kit (QIAGEN), sheared using a Covaris M220 sonicator (Covaris), and size selected prior to library preparation with AMPure XP beads (Beckman Coulter). DNA concentration was determined by Qubit fluorometric quantitation (Thermo Fisher Scientific), and fragment size distribution was assessed by Agilent BioAnalyzer 2100.

CAPP-seq analysis

Briefly, 10ng of purified cfDNA or 20ng of sheared peripheral blood leukocytes (PBL) genomic DNA was used for preparing Illumina-compatible libraries using a modified protocol with the KAPA Hyper Prep Kit (Roche). DNA was end repaired, A-tailed, adapter ligated, and PCR amplified. Hybridization capture was conducted with IDT xGen Lockdown probe sets and xGen Hybridization and Wash kit according to the manufacturer's protocol. Captured libraries were subjected to paired-end sequencing with Illumina NovaSeq 6000. NGScheckMate was used as a quality control (QC) metric to confirm that the plasma sample was from the same subject in the study (1). Mean and median raw depths (including duplicates) were 24926x and 24782x (range 173377-31884x), respectively. Duplex consensus sequences with singleton correction were used for the subsequent variant calling, and all unique molecule sequences were used for the evaluation of VAF by iDES for SNVs (2) and VarDict2 for small insertions/deletions (Indels) (3). To suppress false calls for Indels, we took the

intersection of those called by both Mutect2 and iVarDict2 (at least five supporting reads for Indels).

References for Methods

1. Lee S, Lee S, Ouellette S, Park WY, Lee EA, Park PJ. NGSCheckMate: software for validating sample identity in next-generation sequencing studies within and across data types. *Nucleic Acids Res* 2017;**45**(11):e103 doi 10.1093/nar/gkx193.
2. Newman AM, Lovejoy AF, Klass DM, Kurtz DM, Chabon JJ, Scherer F*, et al.* Integrated digital error suppression for improved detection of circulating tumor DNA. *Nat Biotechnol* 2016;**34**(5):547-55 doi 10.1038/nbt.3520.
3. Lai Z, Markovets A, Ahdesmaki M, Chapman B, Hofmann O, McEwen R*, et al.* VarDict: a novel and versatile variant caller for next-generation sequencing in cancer research. *Nucleic Acids Res* 2016;**44**(11):e108 doi 10.1093/nar/gkw227.

Supplementary Tables

**Characteristics**	**Patients (%)**
**Gender**	
Male	17 (100)
**Stage**	
III HPV+	9 (53)
III HPV-	4 (23.5)
IV A-B	4 (23.5)
**Primary Site**	
Oropharynx	11 (65)
Larynx	3 (18)
Oral Cavity	2 (11)
Hypopharynx	1 (6)
**Papilloma Virus (p16+)**	
Positive	9 (53)
Negative/Not done	8 (47)
**Smoking**	
Never Smoker	4 (24)
Former Smoker	2 (12)
Smoker	11 (64)
**Alcohol Intake**	
Never	5 (29)

Former	1 (6)
Active occasional-moderate	11 (65)
**Treatment**	
Surgery	1 (6)
Surgery followed by adjuvant radiation	1 (6)
Surgery followed by adjuvant chemoradiation	1 (6)
Definitive radiation	2 (12)
Definitive chemoradiation	12 (70)
**Cisplatin**	
Weekly	4 (31%)
High Dose	9 (69%)
Non Applicable	4
**Recurrence**	
Yes	5 (29%)
No	12(71%)

Supplementary Table 1. Patients with bespoke ctDNA analysis (N=17). *HPV: Human papilloma virus*

**Characteristics**	**Patients (%)**
**Gender**	
Male	24 (83)
Female	5 (17)
**Stage**	
III HPV+	15 (52)
III HPV-	6 (21)
IV A-B	8 (27)
**Primary Site**	
Oropharynx	21 (72)
Larynx	5 (17)
Oral Cavity	2 (7)
Hypopharynx	1 (3)
**Papilloma Virus (p16+)**	
Positive	15 (52)
Negative/Not done	14 (48)
**Smoking**	
Never Smoker	6 (20.5)
Former Smoker	6 (20.5)
Smoker	17 (29)
**Alcohol Intake**	
Never	7 (24)
Former	1 (3)
Active occasional-moderate	21 (73)
**Treatment**	
Surgery	1 (3)

Surgery followed by adjuvant radiation	1 (3)
Surgery followed by adjuvant chemoradiation	1 (3)
Definitive radiation	3 (10)
Definitive chemoradiation	23 (79)
**Cisplatin**	
Weekly	10 (42%)
High Dose	14 (58%)
Non Applicable	5

Supplementary Table 2. Patients with baseline sample for HPV-seq and CAPP-seq analysis (N=29). *HPV: Human papilloma virus*

	RaDaR (eVAF)	dPCR HPV (copies/ml)	HPV-seq (copies/ml)	CAPP seq (median VAF)
**Baseline**				
RaDaR	-	0.68	0.60	0.61
dPCR HPV	0.68	-	0.98	0.75
HPV-seq	0.60	0.98	-	0.75
CAPP-seq	0.61	0.75	0.75	-
**FU1**				
RaDaR	-	0.33	0.65	0.15
DPCR HPV	0.33	-	0.60	0.99
HPV-seq	0.65	0.60	-	0.31
CAPP seq	0.15	0.99	0.31	-
**FU2**				
RaDaR	-	0.00	0.63	0.03
dPCR HPV	0.00	-	0.70	1.00
HPV-seq	0.63	0.70	-	0.52
CAPP seq	0.03	1.00	0.52	-

Supplementary Table 3. Spearman correlation among methods at the different timepoints.

	Assay	N	N Relapse	N Positive	N True positive	NPV (%)	PPV (%)	Sensitivity (%)	Specificity (%)	Accuracy (%)
**FU1**	RaDaR™	10	4	0	0	60	-	0	100	60
	CAPP-seq	15	4	2	2	84.7	50	50	100	86.7
	HPV-seq	15	4	3	2	83.3	66.67	50	90.9	80
	dPCR	15	4	2	2	84.62	100	50	100	86.7
**FU2**	RaDaR™	9	3	1	1	75	100	33.3	100	77.8
	CAPP-seq	14	3	1	1	84.6	100	33.3	100	85.7
	HPV-seq	14	3	3	3	100	100	100	100	100
	dPCR	14	3	1	1	84.62	100	33.33	100	85.71
**Any FU**	RaDaR™	10	4	1	1	66.7	100	25	100	70
	CAPP-seq	16	4	2	2	85.7	100	50	100	87.5
	HPV-seq	16	4	5	4	100	80	100	91.67	93.75
	dPCR	16	4	2	2	85.71	100	50	100	87.5

Supplementary Table 4. Assay's performance in detecting MRD in the p16+ oropharyngeal cancer population.

**Timepoint**	**FU1**		**FU2**		**Any FU**	
**Method**	**N**	**2y-RFS % (95%CI)**	**N**	**2y-RFS % (95%CI)**	**N**	**2y-RFS % (95%CI)**
**RaDaR + HPV-seq**<br>• **No detected**<br>• **Detected**	13	86 (63-100)<br>0 (0-0)<br>*P<0.001*	16	88 (67-100)<br>25 (5-100)<br>*P<0.001*	14	83 (58-100)<br>20 (3-100)<br>*P=0.001*
**CAPP-seq + HPV-seq**<br>• **No detected**<br>• **Detected**	24	86 (69-100)<br>40 (9-100)<br>*P=0.037*	25	88 (73-100)<br>53 (21-100)<br>*P=0.014*	21	91 (75-100)<br>43 (15-100)<br>*P=0.002*
**RaDaR + CAPP-seq + HPV-seq**<br>• **No Detected**<br>• **Detected**	13	83 (58-100)<br>25 (5-100)<br>*P=0.06*	16	86 (63-100)<br>40 (14-100)<br>*P=0.007*	14	80 (52-100)<br>33 (11-100)<br>*P=0.02*

Supplementary Table 5. Relapse Free Survival based on multiple assays in FU1, FU2 and any FU. *Patients with a sample not available for analysis were not considered for analysis at the specific follow up while a patient with one FU sample negative but the other not performed, were removed from the analysis at any FU.*

Supplementary Figures

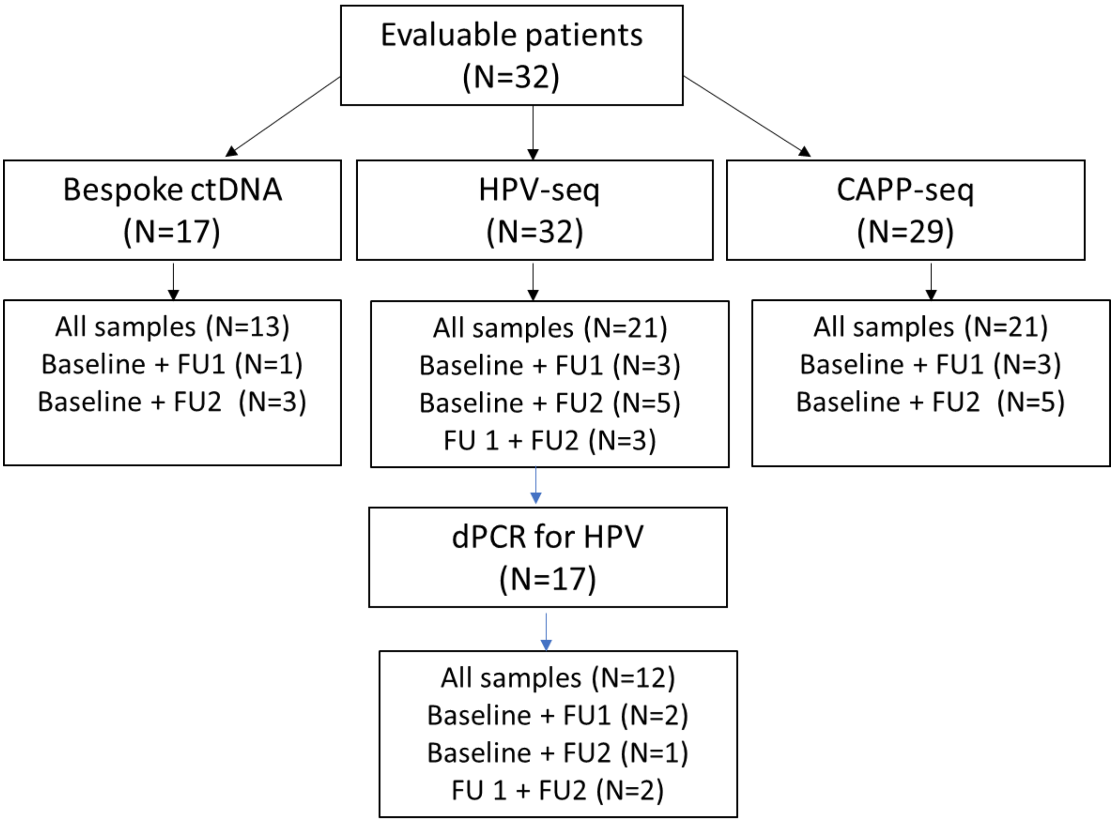

Supplementary Figure 1. Patients enrolled in the PRE-MERIDIAN study and plasma sample availability. CAPP-seq: CAncer Personalized Profiling by deep Sequencing; ctDNA: circulating tumor DNA; FU: follow up; HPV-seq: HPV sequencing;

A

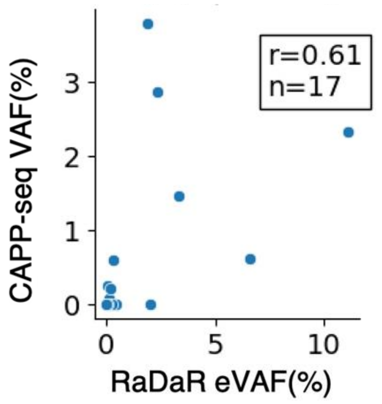

B

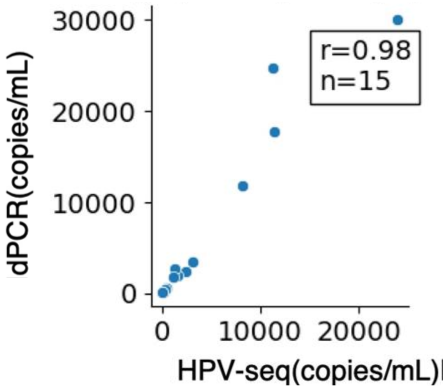

Supplementary Figure 2. Correlation at baseline among the different methods: *A – RaDaR and CAPP-seq; B- HPV-seq and digital PCR.*
